# A delicate balance: Iron metabolism and diseases of the brain

**DOI:** 10.3389/fnagi.2013.00034

**Published:** 2013-07-18

**Authors:** Dominic Hare, Scott Ayton, Ashley Bush, Peng Lei

**Affiliations:** ^1^The Florey Institute of Neuroscience and Mental Health, University of MelbourneVIC, Australia; ^2^Elemental Bio-imaging Facility, University of TechnologySydney, NSW, Australia

**Keywords:** iron regulation, Alzheimer’s disease, Parkinson’s disease, iron deficiency, iron chelation

## Abstract

Iron is the most abundant transition metal within the brain, and is vital for a number of cellular processes including neurotransmitter synthesis, myelination of neurons, and mitochondrial function. Redox cycling between ferrous and ferric iron is utilized in biology for various electron transfer reactions essential to life, yet this same chemistry mediates deleterious reactions with oxygen that induce oxidative stress. Consequently, there is a precise and tightly controlled mechanism to regulate iron in the brain. When iron is dysregulated, both conditions of iron overload and iron deficiencies are harmful to the brain. This review focuses on how iron metabolism is maintained in the brain, and how an alteration to iron and iron metabolism adversely affects neurological function.

## INTRODUCTION

Iron is a fundamental requirement for most known life forms, and is likely to have played an integral role in the earliest development of life on this planet (Russell et al., 1993). Organisms have evolved to harness the unique chemistry of this highly abundant metal, which make it integral to a vast array of chemical reactions supporting cell division, oxygen transport and mitochondrial function. The iron redox couple mediates the transfer of single electrons through the reversible oxidation/reduction reactions of Fe^2^^+^ and Fe^3^^+^. Iron is a *d-*block transition metal, and the unoccupied *d*-orbitals allow ionic iron (II), iron (III), and iron (IV) species to form ligands with both small and large biomolecules via oxygen, nitrogen, and sulfur atoms. The biological redox potential and electronic spin state, and thereby reactivity of iron, is determined by the nature of the ligand to which the species is bound. This configuration, along with the oxidation state of the iron itself, dictates whether an iron-based biomolecule is responsible for reactions involving oxygen transport and storage, electron transfer, or oxidation/reduction of other molecules (Beard, 2001). Reactions involving iron in the body are predominately redox-based, hydrolytic or involve polynuclear complex formation (Aisen, 2001).

Reliance upon iron for normal physiological function has thus necessitated a tightly regulated mechanism for ensuring the net turnover of dietary iron is essentially neutral (Crichton and Ward, 1992). This is especially important for the brain, where some of the highest concentrations of iron in the body are maintained (Gerlach et al., 1994). This review will provide an overview of how brain iron metabolism is regulated, and the consequences of perturbed iron homeostasis.

## IRON UPTAKE, TRANSPORT AND CELLULAR REGULATION

### IRON CIRCULATION AND BRAIN UPTAKE

The major iron transporter protein in the body is the 80 kDa glycoprotein transferrin (Tf). Each bi-lobar molecule, consisting of two globular units at the N- and C-terminals has two iron-binding sites, which form a 4-atom tetradentate ligand via histidine, aspartate, and two tyrosine amino acid residues (Anderson et al., 1987). Almost all iron exchange and transport within the body is mediated by Tf (Finch and Huebers, 1982), with around 3–4 mg of iron typically circulating the healthy adult bound to Tf. Two Fe^3^^+^ ions oxidized by a ferroxidase and shunted into the interstitium by ferroportin are loaded onto a single Tf unit, and at any one time only around 30% of all circulating Tf units are occupied (only in cases of severe iron overload does Tf saturation occur; Aisen, 2001). Less than 1% of circulating iron is usually non-Tf bound. Non-Tf bound iron (NTBI) is handled by a series of low molecular weight (LMW) ligands including citrate and ascorbate ions, as well as a possible small contribution from circulating albumin and ferritin proteins (Breuer et al., 2000) and ATP.

The hydrophobic barricade formed by the blood–brain barrier (BBB) prevents diffusion of hydrophilic Fe_2_Tf into the nervous system, as well as prevent migration of NTBI. Moos et al. (2007) and Crichton et al. (2011) have recently published comprehensive pictures of iron trafficking within the brain, including uptake from the periphery. This step, where Fe_2_Tf is transported across the BBB through brain capillary endothelial cells (BCECs). Tf-uptake into BCECs follows an endocytotic mechanism, where circulating Tf binds to Tf receptors which then internalize. It is a point of contention as to whether iron export from the endosome is mediated by the protein divalent metal transporter-1 (DMT1); conflicting reports have either identified (Burdo et al., 2001) or failed to identify (Moos and Morgan, 2004) DMT1 in rodent brain BCECs. An alternative hypothesis has suggested iron becomes segregated from Tf after liberation from the metal–protein complex in the endosome and is released independently of DMT1 (Moos et al., 2006). During development, when the BBB is not fully formed, there is a rapid influx of iron most likely stemming from NTBI; the developing rat brain shows a rapid intake of iron in line with increased expression of transferrin receptor 1 (TfR1) in BCECs, which in turn becomes the major iron regulatory mechanism once the BBB is sealed, after which iron intake slows (Taylor and Morgan, 1990). However, brain iron import is unlikely solely regulated by BCECs, like many other metabolic pathways redundancies are likely in place in the case one pathway breaks down. For instance, obstruction of BCEC TfR1 in mice and rats using intravenously administered monoclonal antibodies did not completely impede brain iron uptake (Ueda et al., 1993).

A possible alternative mechanism for the uptake of NTBI may be associated with the expression of ferroportin in the BBB (Wu et al., 2004) and circulating ferroxidases (enzymes that catalyze Fe^2^^+^ oxidation to Fe^3^^+^) like ceruloplasmin (Cp; Osaki et al., 1966). It should be noted, however, that expression of ferroportin in BCECs has been disputed (Moos and Rosengren Nielsen, 2006). It is also unclear as to whether iron present in the BCEC endosome is in fact released into the cytosol. Moos et al. (2007) proposed that the possible lack of DMT1 is suggestive that the endosome traverses the BCEC cytosol intact (transcytosis) and releases Fe^3^^+^ directly into the brain for distribution to cells.

On the abluminal side of the BCEC astrocytes abut the cell membrane, forming part of either “neurovascular” or “gliovascular” units comprising of neurons, astrocytes, and BCECs (Abbott et al., 2006). Moos et al. (2007) suggested that astrocyte “end feet” surround the BCEC with a thin layer of interstitial fluid into which iron is released from endosomal Fe_2_Tf–TfR1 complexes on the luminal membrane of the BCEC. Iron is then either re-complexed by Tf in the brain interstitium, or bound to LMW ligands released by the astrocyte. While the affinity of iron to small ligands is considerably smaller than that to Tf, it has been suggested that Tf saturation in the cerebrospinal fluid (CSF) is much higher than in the periphery, and that a larger proportion of NTBI circulates the nervous system (Leitner and Connor, 2012). Astrocytes also provide a source of Cp to ensure any circulating Fe^2^^+^ is quickly oxidized to Fe^3^^+^ to prevent unwanted reactive oxygen species (ROS) production through Fenton chemistry.

### CELLULAR IRON TRAFFICKING IN THE BRAIN

Iron is released from Tf into cells via a particularly elegant mechanism. TfR1 is a ubiquitously expressed membrane protein with a dimeric structure and high affinity to Fe_2_Tf, but at neutral pH, has a low affinity for apo-Tf (iron-free) so that the unligated Tf does not act as a competitive inhibitor of holo-Tf (iron-bound) uptake (Aisen, 2004). The Fe_2_Tf forms a complex with the TfR1 receptor, which is then endocytosed. A proton pump mechanism is initiated to lower the pH within the endosome, which causes a conformational change to both the Fe_2_Tf and TfR1 units, in turn resulting in release of the iron from its chaperoning protein (Hentze et al., 2004). The newly freed Fe^3^^+^ is quickly reduced by the six-transmembrane epithelial antigen of prostate 1-4 (STEAP 1-4), allowing export from the endosome into the cytosol by DMT1 (De Domenico et al., 2008). In the acidic endosome, apo-Tf has a strong affinity for the Tf receptor; this interaction prevents the degradation of free Tf when the endosome complexes with the lysosome before exocytosis. During exocytosis the pH returns to neutral, which causes dissociation of the apo-Tf from the TfR1, effectively recycling the Tf molecule for further use in iron circulation (Dautry-Varsat et al., 1983). In 1999, a homolog Tf receptor, TfR2, was identified (Kawabata et al., 1999), which initially showed expression only in hepatocytes, duodenal crypt cells, and erythrocytes. TfR2 has a 30-fold lower affinity to iron-bound Tf, yet mutations to the TfR2 gene results in hereditary hemochromatosis (Camaschella et al., 2000). TfR2 shares 45% amino acid identity with the ubiquitous TfR1 (Kawabata et al., 1999; Fleming et al., 2000). Interestingly, TfR2 has also been identified in dopaminergic neurons, and has been suggested to play a role in Fe_2_Tf translocation to mitochondria (Mastroberardino et al., 2009).

**FIGURE 1:**
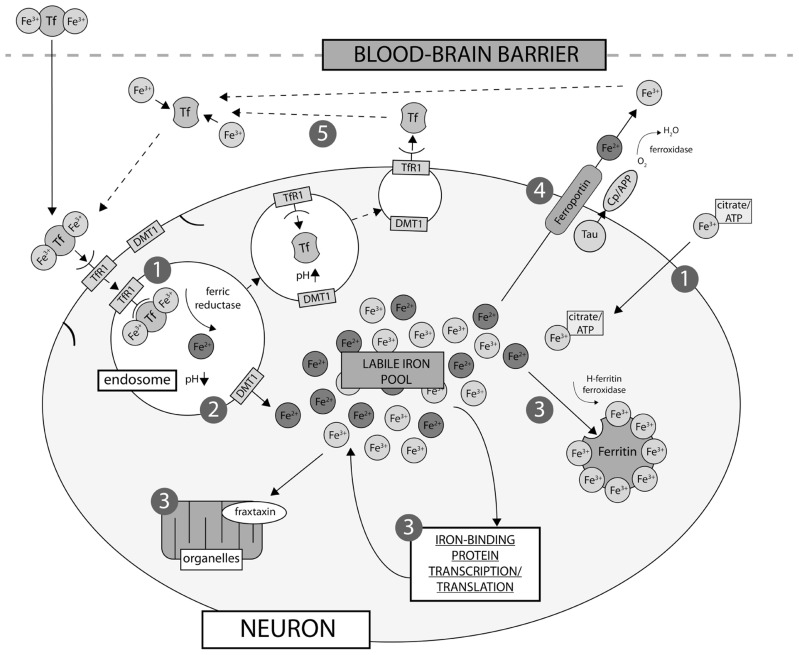
**Neuronal iron uptake and export.** Iron is imported into the cell either as low molecular weight complexes with citrate/ATP, or via transferrin receptor (TfR1)-mediated endocytosis (1). Export of iron from the endosome via divalent metal transporter-1 (DMT-1) directly contributes to the labile iron pool (2), which constitutes the available iron content of the neuron and is regulated by cellular metal sensing and iron-binding protein expression, including ferritin, which is the major iron storage protein in neurons (3). Export of iron from neurons is regulated by intramembrane ferroportin, which is stabilized via a mechanism involving tau and ceruloplasmin (Cp)/APP (4). Iron is then recirculated by apo-Tf (5).

Neurons express both TfR1 and DMT1 (Burdo et al., 2001), and therefore uptake iron via a receptor-mediated endocytotic mechanism (**Figure 1**), though it is likely that a small minority of iron uptake is sourced from NTBI *in vivo*. Astrocytes are devoid of TfR1, and NTBI is most likely their major iron source (Moos and Morgan, 2004). Oligodendrocytes, which require iron for myelin synthesis (see below; Connor and Menzies, 1998) also import iron through a mechanism independent of TfR1. Two noteworthy hypotheses have been proposed to explain how the comparatively high need for iron by oligodendrocytes is regulated without the major iron import mechanism present. Firstly, iron passes into the cytosol complexed with LMW ligands. Iron is then incorporated into Tf produced within the oligodendrocyte itself, where it is either used immediately or sequestered in ferritin for storage (Moos et al., 2007). Tf is not secreted by the oligodendrocyte itself (de Arriba Zerpa et al., 2000), presenting a fairly unique closed environment of iron regulation in what is predominately otherwise an intertwined regulatory system.

Once inside the cell, iron can follow multiple pathways dependent on need. Ferritins are responsible for iron storage and play an integral role in iron homeostasis, and are rarely saturated due to their large capacity for thousands of individual Fe^3^^+^ ions (Theil, 2004). Numerous other cytosolic proteins require iron for a variety of normal functions. Iron is also important to mitochondrial functions, where it is incorporated into Fe–S clusters and heme proteins (Hentze et al., 2004). The mechanism for mitochondrial uptake has not been categorically confirmed, though the two proposed pathways involve either (i) diffusion of NTBI or (ii) direct translocation of extracellular Fe_2_Tf via an endosomal pathway (Horowitz and Greenamyre, 2010). Within the mitochondria, the frataxin protein (implicated in Friedreich’s ataxia) is suggested to act as a intramitochondrial iron chaperone (Richardson et al., 2010).

The only known export pathway in mammalian cells is mediated via ferroportin (Ganz, 2005). Ferroportin allows ferrous iron to be transported out of the cell (Donovan et al., 2005), and this process requires a ferroxidase to oxidize the ferrous iron to ferric, so that Tf can bind the exported iron. In the brain, ferroportin has been identified in both neurons (Abboud and Haile, 2000) and astrocytes (Dringen et al., 2007), as have the corresponding ferroxidases, the amyloid precursor protein (APP; Duce et al., 2010) and Cp (Texel et al., 2011).

### REGULATION OF IRON-ASSOCIATED PROTEINS

**FIGURE 2:**
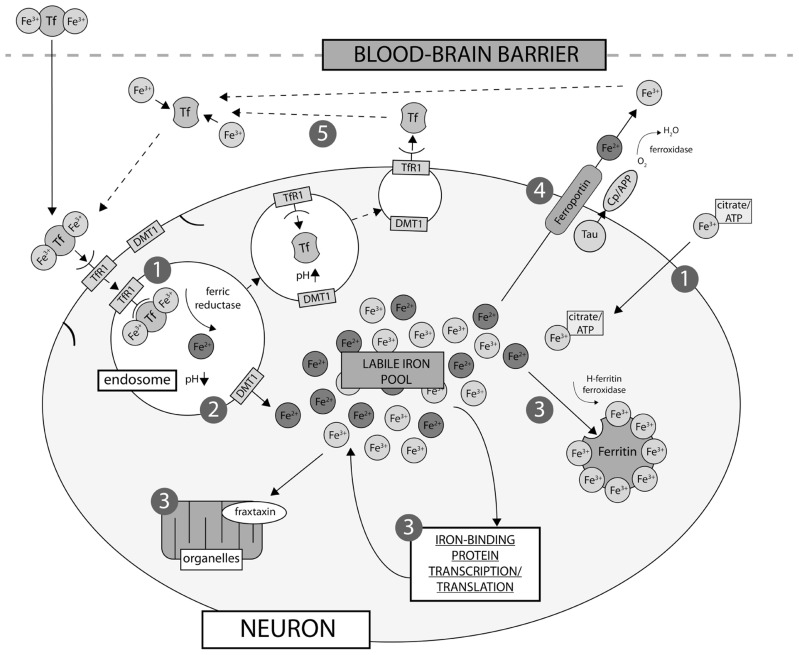
**Factors influencing cellular iron metabolism.** Cellular oxygen homeostasis regulates iron metabolism in the nucleus via the hypoxia inducible factors (HIF-1α and -1β). In normoxia conditions, Fe^2^^+^ mediates the hydroxylation of proline residue 546 on HIF-1α by prolyl-4-hydroxylase (PHD), which enables ubiquitination via binding with the von Hippel–Lindau tumor suppressor gene product (VHL). Decreased HIF-1α inhibits transcription of proteins dictating iron uptake and export. In the iron-deficient cell, HIF-1α instead interacts with CREB binding protein/p300 (CBP/p300) and forms a heterodimer with HIF-1β, activating transcription of target genes possessing hypoxia response elements. In the cytosol, the iron replete cell prevents the binding of iron responsive proteins (IRP) -1 and -2 to the iron responsive elements (IREs) in the 5′ and 3′ untranslated region (UTR) of mRNA, inhibiting the transcription of uptake proteins and promoting expression of proteins involved in iron export. In cases of iron deficiency, IRPs directly interact with the 5′- and 3′-UTRs, eliciting the reverse effect. TfR, transferrin receptor; DMT-1, divalent metal transporter-1; APP, amyloid precursor protein.

Iron-associated proteins are regulated by iron status, therefore form a cycle to regulate iron metabolism (**Figure 2**). In cases of low cellular iron, two iron regulatory proteins (IRP1/2) are free to bind directly with iron responsive element (IRE) stem-loop structures within the mRNA of iron-binding proteins. The 3′ untranslated portion of, for example, TfR1 mRNA is sensitive to ribonuclease degradation, thus binding with IRP1/2 protects the mRNA and promotes TfR1 expression, increasing cellular iron uptake. Conversely, binding of IRP1/2 to the 5′ untranslated region (UTR) of, for example, ferritin mRNA prevents translation, reducing cytoplasmic ferritin expression, reducing the iron storage capacity of the cell, and increasing available iron (Aisen, 2001). When iron levels in the cell are high, labile iron binds with IRP1/2, preventing interactions between the regulatory proteins and the IREs in the mRNA of various iron regulating proteins (see **Figure 2**), eliciting the reverse cellular response to that observed in cases of iron deficit. The mechanism of iron-mediated inhibition of IRP/IRE binding depends on the protein involved: IRP1–Fe undergoes a conformational change that prevents IRE binding, whereas IRP2–Fe complexes undergo degradation via the ubiquitin proteasome pathway (Pantopoulos, 2004). Both IRP1 and IRP2 are present in the rat (Siddappa et al., 2003) and human brain (Connor et al., 1992c); IRP1 has been suggested as the primary regulatory protein in the human brain and is capable of forming a double IRP1/IRE complex (Hu and Connor, 1996).

Iron metabolism is also transcriptionally regulated by the action of hypoxia inducible factors (HIFs; see **Figure 2**), which consists of a cytosolic protein (HIF-1α) and a nuclear HIF-1β subunit that form a DNA binding heterodimer (Peyssonnaux et al., 2008). HIF-1α levels are dictated by cellular chemistry, and in normal conditions HIF-1α is hydroxylated by prolyl hydroxylase, marking the adduct for ubiquitination and degradation (Ratcliffe et al., 1999). This reaction requires oxygen, 2-oxoglutarate, ascorbate, and iron as cofactors. In hypoxic conditions HIF-1α ubiquitination is inhibited and translocation to the nucleus is increased, where dimerization with HIF-1β allows binding with cyclic adenosine monophosphate (cAMP) response element-binding protein (CREB), which activates transcription of target genes with hypoxia response elements (HREs; Semenza, 2000). As iron is necessary for hydroxylation of HIF-1α, any decrease in cellular iron levels will increase dimerization of HIF-1α and β and downstream transcription of target genes, which, unsurprisingly, includes those responsible for Tf (Rolfs et al., 1997), TfR1 (Lok and Ponka, 1999), and DMT1 (Lis et al., 2005).

With such a sophisticated regulation mechanism for regulating iron-associated proteins transcription and translation, it is surprising diseases of iron overload and deficiencies exist. Some of these disorders arise from nutritional deficiency/excess or genetic mutation in one or more iron-associated proteins. But as will be described, the disorders of iron metabolism are invariably chronic disorders, not acute disorders. Possibly because there is complex regulatory mechanism that can resist, and then compensate for, short-term changes to iron levels.

## IRON FLUX AND DEFICIENCY

Iron deficiency is a major nutrition deficiency most often observed in the developing world. Even in a developed nation, such as the USA, approximately 10% of toddlers and women of childbearing age are iron-deficient (Looker et al., 1997). As described above, the cellular iron regulatory mechanism is nuanced and robust, therefore allowing considerable divergence from the homeostatic norm of iron levels before disease precipitates. Disorders of iron homeostasis are thus invariably chronic, not acute diseases. This is especially true of the brain, which has more stable iron levels compared to the other organs (Youdim et al., 1989).

Peripheral iron deficiency can result in minor to severe symptoms. Anemia is an advanced iron deficiency syndrome caused by a number of factors ranging from dietary deficiency, blood loss, and metabolic lesions. The most common symptoms arising from anemia result from reduced oxygen transportation by hemoglobin. These symptoms include pallor, fatigue, faintness, shortness of breath, muscle weakness, angina pain, and elevated cardiac output (as compensation for reduced oxygen carrying capacity; Wood and Elwood, 1966).

While symptoms of anemia can arise over a period of weeks to months, iron in the brain is more resistant to dietary changes (Youdim et al., 1989) and indeed the brain may have critical periods that determine the level of iron in the brain throughout life (Dallman et al., 1975; Dallman and Spirito, 1977; Ben-Shachar et al., 1986). The brain accumulates iron during the weaning period, which establishes an iron “set point” for the brain. Perinatal pups on a restricted iron diet cannot recover their brain iron levels when supplemented later in life (Ben-Shachar et al., 1986). It has been proposed that the brain of a post-weaned mammal is impermeable to peripheral iron (Kaur et al., 2007), possibly explaining why brain iron levels are not easily altered with diet (Ben-Shachar et al., 1986). However, supplementation of isotopically enriched iron to mice reveals dietary iron incorporation into the brain at a similar rate to other organs (Chen et al., 2013). Peripheral iron is able to enter the brain by patterning with Tf, which can undergo receptor-mediated transcytosis to pass through the BBB (Fishman et al., 1987; Bradbury, 1997; Morgan and Moos, 2002). It is likely that brain iron is in constant flux with peripheral pools of iron. We speculate that brain iron levels do not tangibly deviate from the norm despite changes in the diet, because the brain iron levels are strictly governed by the iron homeostatic mechanism, and where the normal level of iron in the brain for an adult individual is likely determined in the critical weaning period of the mammal. This period can thus shape the iron biochemistry of the brain throughout the life of an individual, highlighting the urgency of addressing nutritional deficiency in infants raised in areas of poverty. Described below, chronic brain iron deficiency disrupts important process in the brain, altering neurochemistry that eventually leads to disease.

## NEUROCHEMICAL EFFECT OF IRON DEFICIENCY

### NEUROTRANSMITTER SIGNALING

Iron affects synthesis and signaling of the neurotransmitters dopamine, noradrenalin, adrenaline and 5-hydroxytryptamine, which are involved in emotion, attention, reward, movement, and various other functions. These neurotransmitters are synthesized by a number of iron-dependent enzymes including phenylalanine hydroxylase (Gottschall et al., 1982), tyrosine hydroxylase (Ramsey et al., 1996), and tryptophan hydroxylase (Kuhn et al., 1980). Brain iron deficiency (BID), however, rarely causes reduced expression or activity of these enzymes (Youdim et al., 1989). The conservation of iron in these enzymes under BID possibly reflects the importance of these enzymes to brain function.

In addition to neurotransmitter synthesis, iron impacts several other steps in neurotransmitter signaling, which are more vulnerable to changes in iron levels. Reduced neuronal uptake of the catecholaminergic neurotransmitters has been observed in several BID models (Burhans et al., 2005; Beard et al., 2006b; Bianco et al., 2008), and the extracellular concentration of neurotransmitters are elevated in BID rats (Beard et al., 1994). Dopaminergic signaling is further perturbed in iron deficiency by attenuating affinity and expression of D2 neurotransmitters (Youdim et al., 1989).

### ENERGY PRODUCTION

The brain has a high energy demand, accounting for 20% of basal oxygen consumption (Halliwell, 2006) and thus requires high iron levels to generate ATP by the electron transport chain in the mitochondria. Various mitochondrial enzymes utilize iron as a cofactor including the mitochondrial ferredoxins (Redfearn and King, 1964), cytochromes (Slater, 1949), and aconitase (Dickman and Cloutier, 1950). Iron deficiency changes mitochondria morphology (Jarvis and Jacobs, 1974), impairs function (Masini et al., 1994), and damages mitochondrial DNA (Walter et al., 2002). Reduced mitochondrial efficiency possibly explains why iron deficiency results in elevation of oxidative stress markers (Knutson et al., 2000; Jeong et al., 2011; Wan et al., 2012a), despite loss of pro-oxidant iron.

### MYELINATION

Myelin is the fatty “white matter” that insulates axons and preserves their signaling. Accordingly, under basal conditions, oligodendrocytes exhibit high levels of iron comparative to other brain cells (Benkovic and Connor, 1993; Connor et al., 1995). Iron treatment to cultured glial restricted precursor cells increases their differentiation into GalC1 oligodendrocytes, while treatment to cultured O2A oligodendrocytes progenitors increases their proliferation without altering differentiation (Morath and Mayer-Proschel, 2001). Iron, therefore, has marked, but distinct effects on the temporal sequence of oligodendrocyte development. In a rat model, BID restricts both glia precursor cell proliferation and differentiation into oligodendrocytes (Morath et al., 2002) and decreases components of myelin: myelin basic protein, myelin proteolipid protein, galactolipids, phospholipids, and cholesterol (Yu et al., 1986; Ortiz et al., 2004). Lack of myelination causes slower neuronal conduction, evidenced by retardation of reflexes. In humans, iron deficiency is associated with abnormal reflexes in infants (Armony-Sivan et al., 2004) and in iron-deficient children, deficits in auditory brain stem potentials and visual evoked potentials have been observed (Roncagliolo et al., 1998; Algarin et al., 2003).

## NEUROLOGICAL DISORDERS ASSOCIATED WITH IRON DEFICIENCY

### FAILURE TO THRIVE

It is now widely recognized that BID in early life is associated with developmental delays in various brain faculties. Iron deficiency, characterized by anemia, has been associated with poorer fine and gross motor skills, visual-motor integration, language and global IQ, accompanying higher scores in anxiety and depression, social and attention problems (Palti et al., 1985; Lozoff et al., 1991; Hurtado et al., 1999) with some symptoms persisting 10 years after treatment for anemia (Lozoff et al., 2000). While the association between iron and various markers of developmental delay are unequivocal, the causal relationship is complicated by confounding socioeconomic variables that often accompany iron deficiency including generally poor nutrition, lack of stimulation in the home, lack of maternal warmth, poor maternal education, maternal depression, more absent fathers, parasitic infection, and low birth weight (Grantham-McGregor and Ani, 2001).

The importance of iron to neurodevelopment is thus unclear from observational human studies, which has necessitated study of iron-deficient experimental animal models. Agreeing with complementary human studies, BID in rats causes delayed behavioral milestones (Beard et al., 2006a), including impaired memory (Yehuda et al., 1986; Wachs et al., 2005) and motor function (Hunt et al., 1994). Symptoms resulting from dietary iron restriction in the first 21 days of the life of the rat are not recoverable even after 6 weeks of iron supplementation (Ben-Shachar et al., 1986). Combining the human and animal evidences strongly supports a critical role for iron in neurodevelopment, and since the symptoms are not readily correctable after the critical period, these also highlight the importance of monitoring and early dietary intervention.

### ATTENTION DEFICIT HYPERACTIVITY DISORDER

Attention deficit hyperactivity disorder (ADHD) is a developmental disorder manifesting in symptoms of inattention, hyperactivity, and impulsiveness. ADHD is highly heritable, and several candidate disease-causing genes are involved in dopamine neurotransmission (*DAT1, DRD4, DRD5*; Elia and Devoto, 2007)*. *Since iron interacts with multiple steps in dopamine neurotransmission, it is possible that BID might precipitate ADHD in idiopathic cases. While several studies showed reduced ferritin in children affected by ADHD (Konofal et al., 2004; Oner et al., 2008; Cortese et al., 2009; Juneja et al., 2010; Menegassi et al., 2010), the largest study (194 children), reported unaltered serum ferritin levels between ADHD patients and controls (Donfrancesco et al., 2012). As previously mentioned, iron status in children often co varies with multiple parameters of socioeconomic status, which might confound these studies. Further peripheral markers of iron do not often reflect the status of brain iron; therefore peripheral iron is not likely altered in ADHD. However, a recent study of 36 individuals reported reduced brain iron in the thalamic region as measured by magnetic resonance imaging (MRI) in ADHD patients (Cortese et al., 2012) suggesting a role for BID in the pathogenesis of this disease.

Could iron supplementation therefore be used as a treatment for ADHD? A case study reported a 3 year-old presenting with low serum ferritin (13 ng mL^-^^1^) accompanying ADHD who was supplemented with ferrous sulfate (80 mg day^-^^1^) and 8 months later was observed to exhibit various behavioral improvements (Konofal et al., 2005). This prompted a 12-week clinical trial of iron supplementation in ADHD, which recorded improvements in the ADHD rating scale for the treatment group (Konofal et al., 2008). These studies warrant further investigation into iron as a potential therapeutic, however, as discussed above, iron supplementation after a critical period is not effective in reversing cognitive symptoms of early BID in rats (Ben-Shachar et al., 1986), which might limit the use of this approach in ADHD.

### RESTLESS LEGS SYNDROME

Restless legs syndrome (RLS) is a neurological disorder characterized by uncomfortable or odd sensations in the body (often legs) that prompt an incessant urge to move (Earley, 2003). The prevalence of RLS is estimated to be between 5 and 10% of the population (Lavigne and Montplaisir, 1994; Rothdach et al., 2000; Ulfberg et al., 2001). The disorder is associated with reduced dopamine uptake and reduced D2 receptor density (Staedt et al., 1995; Turjanski et al., 1999; Michaud et al., 2002), and is often treated with dopamine-based therapies (Hening et al., 1999; Allen et al., 2001). This neurochemical profile is consistent with BID (Youdim et al., 1989) of the nigrostriatal pathway. Indeed low ferritin and high Tf levels have been reported in CSF of RLS patients, while serum indices of iron metabolism were not altered (Earley et al., 2000; Clardy et al., 2006). Direct measurements of iron by post-mortem histological staining (Connor et al., 2003) and MRI (Allen et al., 2001; Earley et al., 2006) reveal decreased levels in the substantia nigra (SN) of affected patients.

### NEURODEGENERATION

What are the lifetime consequences of BID? This remains an underexplored subject in brain iron research, which has historically focused on BID in neurodevelopment, and iron accumulation in neurodegeneration. Accordingly, to our knowledge, there has been no report of BID in a neurodegenerative disorder. Recently, a genetic mouse model of motor neuron iron deficiency (IRP2^-^^/^^-^) exhibited reduced mitochondrial activity, hypomyelination, and neurodegeneration (Jeong et al., 2011), raising the possibility of BID-induced neurodegenerative disorders. Patients with the neurodegenerative disorder, dementia with Lewy bodies (DLB), have a threefold higher incidence of self-reported history of ADHD symptoms. However, the status of iron in ADHD patients is only beginning to emerge, and the status of iron in DLB is also not known, so it is premature to mechanistically connect the two diseases via iron. Low iron levels impair mitochondrial function (Masini et al., 1994), and increase oxidative stress markers (Knutson et al., 2000), possibly by limiting the function of the iron-dependent antioxidant, catalase (Wan et al., 2012a). Longer-term studies of rodent models of BID will illuminate the neuroanatomical and neurobiochemical changes that result from low iron bioavailability.

## IRON ACCUMULATION IN THE BRAIN

The sophisticated mechanisms that manage iron in the brain highlight the need for tightly controlled iron regulation, in order to exploit its utility in cellular operations, while preventing its deleterious capacity. Functional loss of IRPs by genetic mutations induces brain iron deposition, which is sufficient to cause neurodegeneration in diseases like aceruloplasminemia (Miyajima et al., 1987; Hochstrasser et al., 2004) and neuroferritinopathy (Feyt et al., 2001; Chinnery et al., 2007). This demonstrates the potential for iron elevation to participate in neuronal loss of more common neurodegenerative diseases [e.g., Alzheimer’s (AD) and Parkinson’s disease (PD)] where brain iron elevation features in both diseases.

### BRAIN IRON ACCUMULATION WITH AGING

Aging is an important risk factor for neurodegenerative diseases. Multiple failures of the iron regulatory system in disease could be contributed to by the aging process (Bartzokis et al., 1997; Martin et al., 1998; Pfefferbaum et al., 2009; Penke et al., 2012; Daugherty and Raz, 2013). Age-related iron retention can serve as predictors of behavioral deficits, such as cognitive decline (Penke et al., 2012) and motor impairment (Cass et al., 2007; Kastman et al., 2012), highlighting the possibility of its involvement in age-associated decline.

Brain iron elevation with age could be contributed to by changes in various proteins that comprise the iron regulation machinery. Ferritin is elevated during the aging process in both gray and white matter of occipital cortex (Connor et al., 1992b) and the SN (Zecca et al., 2004), but is unchanged in motor cortex and superior temporal gyrus (Connor et al., 1992b). Tf expression was found to be decreased in white matter of superior temporal gyrus, but elevated in white matter of occipital cortex (Connor et al., 1992b). Cp was found to be elevated in gray matter with aging, without changes in white matter (Connor et al., 1993), while another report observed that Cp was unchanged in SN (Zecca et al., 2004). In rat brains, iron and ferritin were found to increase with age, while Tf levels remain unchanged (Roskams and Connor, 1994). The mechanism of age-related iron accumulation is only beginning to be elucidated. The selective vulnerability of iron accumulation during aging could also explain why iron elevation is a feature of various neurodegenerative diseases.

### ALZHEIMER’S DISEASE

Alzheimer’s disease is the most prevalent neurodegenerative disease characterized clinically by progressive dementia, and pathologically by the presence of Aβ-containing plaques, and tau-containing neurofibrillary tangles in affected brain areas. Elevated iron is also a feature of AD-effected post-mortem brains (Zhu et al., 2009; Duce et al., 2010; Smith et al., 2010; Qin et al., 2011; Antharam et al., 2012; Loef and Walach, 2012). Iron accumulation occurs in AD cortex and hippocampus, but not cerebellum (Andrasi et al., 1995; Duce et al., 2010; Antharam et al., 2012), consistent with the pathological profile of neurodegeneration in AD. In addition, iron is accumulated in both plaques and tangles (Connor et al., 1992a; Smith et al., 1997; Meadowcroft et al., 2009), and is estimated to be three times that of the normal neuropil level in plaques (Lovell et al., 1998). The iron content in hippocampus of patients with AD was reported to correlate with the mini-mental state examination (MMSE) and the disease duration (Ding et al., 2009; Zhu et al., 2009), suggesting that iron can play a significant role in the disease progression.

Several genes of iron regulatory proteins are risk factors for sporadic AD, including Tf and human hemochromatosis protein (HFE). In a genome-wide association study (GWAS) study, Tf variant C2 positively correlates with AD risk with an OR of 1.21 (Bertram and Tanzi, 2008), which is supported by a number of independent studies (Van Landeghem et al., 1998; Schjeide et al., 2009; Kauwe et al., 2010) but was not confirmed in a recent large-scale GWAS study (Hollingworth et al., 2011). In addition, HFE mutations (H63D and C82Y) are risk factors for AD independently (Sampietro et al., 2001; Blazquez et al., 2007), and synergistically with *APOE* gene (Kauwe et al., 2010; Giambattistelli et al., 2011; Lehmann et al., 2012). Both of the genes are also shown to modulate iron content, and are implicated in the risk of cognitive impairment in normal aging (Bartzokis et al., 2011).

Iron accumulation can promote aggregation of both Aβ and tau, the key proteins involved in plaque and tangle formation, respectively. Three histidine residues of Aβ were suggested as the binding amino acids of iron, and this complex is redox-active (Nakamura et al., 2007; Bousejra-ElGarah et al., 2011). Recently it was found that iron delayed the amyloid fibril formation but enhanced the toxicity *in vitro*, suggesting the iron-bound Aβ oligomer could serve as a toxic species (Mantyh et al., 1993; Schubert and Chevion, 1995; Liu et al., 2011). These observations are relevant to disease since iron is concentrated in plaques (Meadowcroft et al., 2009; Gallagher et al., 2012), and increased iron content is prior to plaque formation in an animal model of AD (Leskovjan et al., 2011). Aβ–iron complex can induce ROS via Fenton chemistry (Rottkamp et al., 2001; Rival et al., 2009), and activate B-cell lymphoma 2 (Bcl-2) apoptosis pathway (Kuperstein and Yavin, 2003). Chelation of iron can prevent Aβ aggregation, and reverse the consequent memory loss in animal models of AD (Huang et al., 2004; Guo et al., 2013b).

Iron and tangles co-localized in AD (Smith et al., 1997) and tangles can bind iron in a redox-dependent manner, acting as a source for ROS within the neurons (Smith et al., 1997; Sayre et al., 2000). This process can also be removed by iron chelation (Shin et al., 2003). Fe(III), but not Fe(II), can induce tau aggregation *in vitro*, which again can be reversed by reducing Fe(III) to Fe(II) (Yamamoto et al., 2002) or iron chelators (Amit et al., 2008). Fe(II) can induce tau hyperphosphorylation (Lovell et al., 2004; Chan and Shea, 2006), via activation of extracellular signal-regulated kinase 1/2 (Erk1/2) pathway or the mitogen-activated protein kinase (MAPK) pathway (Muñoz et al., 2006; Huang et al., 2007). Chelation therapies such as deferoxamine can inhibit iron-induced tau hyperphosphorylation *in vivo *(Guo et al., 2013a), and prevention of iron uptake can also inhibit this event by deactivating glycogen synthase kinase 3 (GSK-3) and cyclin-dependent kinase 5 (Cdk-5; Xie et al., 2012), two key tau kinases (Lei et al., 2011).

Understanding the cause of iron accumulation in AD might lead to new therapeutic opportunities. Multiple components of the iron regulatory system are altered in AD including IRP2 (Smith et al., 1998), ferritin (Connor et al., 1992a), and Tf (Loeffler et al., 1995). Of particular interest is APP, which is involved in Aβ production and iron homeostasis. APP expression is unchanged in AD cortex but its ferroxidase activity was reported to be decreased (Duce et al., 2010). This could prevent ferroportin-mediated iron export and Tf loading, which would lead to iron retention within neurons. APP-mediated iron export is also impacted by AD-associated tau protein (Lei et al., 2012). Soluble tau levels are reduced in AD brains compared to control brains (Ksiezak-Reding et al., 1988; Shin et al., 1992; Khatoon et al., 1994; Zhukareva et al., 2001, 2003; van Eersel et al., 2009); which might result from tau deposition into insoluble aggregates during the disease progress (Khatoon et al., 1994). Loss of functional tau could further perturb APP-mediated iron export by restricting the presentation of APP at the surface.

### PARKINSON’S DISEASE

Parkinson’s disease is the most prevalent movement disorder, caused by loss of dopaminergic neurons in the SN pars compacta. The brain of PD patients, and especially the SN, is decorated by Lewy body inclusions that are enriched with the α-synuclein protein. As early as 1924, iron deposition in the SN of PD patients was described (Lhermitte et al., 1924). Iron elevation within this nucleus has been consistently reported using multiple techniques such as inductively coupled plasma-mass spectrometry (ICPMS; Dexter et al., 1989; Lei et al., 2012), atomic absorption spectroscopy (Ayton et al., 2012b), X-ray fluorescence (Popescu et al., 2009), and MRI (Bartzokis et al., 1999).

Iron deposits have been found in Lewy Bodies in PD cases (Castellani et al., 2000), suggesting that α-synuclein may interact with iron at the biochemical level. Indeed, iron binds to α-synuclein (Bharathi and Rao, 2008; Peng et al., 2010), accelerates α-synuclein aggregation (Golts et al., 2002; Kostka et al., 2008), and causes toxic hydroxyl radical production *in vitro* (Turnbull et al., 2001). Treating iron to cells initiates α-synuclein aggregation (Ostrerova-Golts et al., 2000; Gault et al., 2010; Li et al., 2011), and the resultant oligomer promoted α -amino-3-hydroxy-5-methyl-4-isoxazolepropionic acid (AMPA)-receptor-mediated excitotoxicity (Huls et al., 2011). Iron administration to cells that overexpress a disease-related mutant form of α-synuclein, A53T, enhanced cytotoxicity of the protein via increasing the autophagic activity (Ostrerova-Golts et al., 2000; Chew et al., 2011), which could explain how iron causes toxicity in PD.

Iron content in SN is a risk factor and may serve as a biomarker of PD. Mutations in a number of iron-related proteins have been shown to associate with the risk of PD, including Tf (Borie et al., 2002), IRP2 (Deplazes et al., 2004), ferritin (Foglieni et al., 2007), and DMT1 (He et al., 2011). It has been debated whether iron accumulation in SN is a secondary effect of cell death in PD. However, recent developments in MRI and transcranial sonography (TCS) makes it possible to examine brain iron content in living patients. It has been shown using MRI that iron accumulates at the early stage of PD before the symptom onset (Bartzokis et al., 1999; Martin et al., 2008), and healthy individuals with increased SN iron content determined by TCS had 17 times higher risk of developing PD (Berg et al., 2011). The SN iron elevation in PD patients, shown by MRI, correlates with the disease susceptibility (Baudrexel et al., 2010), severity (Atasoy et al., 2004; Wallis et al., 2008) and duration of the disease (Kosta et al., 2006; Zhang et al., 2010). The early rise in iron, measured by TCS and MRI supports a role for iron in the pathogenicity of PD.

Iron accumulation is alone sufficient to cause parkinsonian neurodegeneration. Direct iron injection to rat brains can cause SN neuron loss (Ben-Shachar and Youdim, 1991), and feeding neonatal mice with iron can trigger later life parkinsonism and nigral degeneration (Kaur et al., 2007). Diseases primarily characterized by brain iron accumulation, including aceruloplasminemia (Miyajima et al., 1987; Hochstrasser et al., 2004; McNeill et al., 2008), neuroferritinopathy (Crompton et al., 2002; Chinnery et al., 2007), and iron accumulation (NBIA) (Schneider et al., 2012), often cause symptoms of PD. The observations from these diseases which are caused by rare loss-of-function mutations of IRPs indicate that a similar iron accumulation observed in idiopathic PD likely participates in the degenerative processes. Aceruloplasminemia can be recapitulated in mice that lack the Cp gene, and this can be rescued with iron chelation (Patel et al., 2002; Ayton et al., 2012b).

Modulation of iron shows beneficial effects on PD animal models. PD toxin model, 1-methyl-4-phenyl-1,2,3,6-tetrahydropyridine (MPTP) or 6-hydroxydopamine (6-OHDA) cause SN iron accumulation in mice, coincident with neuronal loss (Hare et al., 2013). These PD models can be rescued by iron chelation (Kaur et al., 2003; Mandel et al., 2004; Youdim et al., 2004a, b). Iron-mediated toxicity in these models can also be ameliorated by genetic or pharmacologically restoring ferritin (Kaur et al., 2003) and Cp (Ayton et al., 2012b).

Why does iron accumulate in PD? This could be contributed by a number of iron-related proteins that are changed in PD. Ferritin levels have been found to be decreased in post-mortem PD brains (Dexter et al., 1990; Werner et al., 2008); loss of iron storage capacity potentially makes free iron species more available for toxic interactions. Iron accumulation in PD might be caused by increased neuronal iron import. DMT1 is elevated in SN of PD patients (Salazar et al., 2008), which could promote iron import, but the levels of TfR1, which is required for DMT1-mediated iron import are unchanged when corrected for neuronal loss (Mash et al., 1991; Morris et al., 1994; Faucheux et al., 1997). Alternatively, iron accumulation in PD could also be attributed to reduced iron export. Cp levels in PD brains were unaltered, however, the activity is selectively reduced in SN, which could bottleneck iron export (Ayton et al., 2012b). Tau protein is also implicated in PD (Lei et al., 2010), and selective reduction of tau found in SN of PD patients may also contribute to iron accumulation by preventing APP-mediated iron export (Lei et al., 2012).

### OTHER NEUROLOGICAL DISORDERS

Iron accumulation has been observed in affected brain regions of various diseases including progressive supranuclear palsy (Coffey et al., 1989; Dexter et al., 1991; Boelmans et al., 2012), Pick’s disease (Ehmann et al., 1984), Huntington’s disease (Dexter et al., 1991; Bartzokis et al., 2007; Jurgens et al., 2010; Rosas et al., 2012), prion disorders (Singh et al., 2009a, 2012), amyotrophic lateral sclerosis (Oba et al., 1993; Santillo et al., 2009; Langkammer et al., 2010), and multiple system atrophy with striatonigral degeneration (Dexter et al., 1991; Vymazal et al., 1999; von Lewinski et al., 2007; Wang et al., 2012). The iron accumulation in diseases such as prion disorders and Huntington’s disease may result from ferritin accumulation (Simmons et al., 2007; Singh et al., 2012), but the cause or implications of iron elevation for these diseases is unclear at this stage.

Friedreich’s ataxia is a disorder of iron metabolism more extensively studied. This autosomal recessive degenerative disease results from mutations in the mitochondrial protein frataxin (Campuzano et al., 1996; Carvajal et al., 1996). Friedreich’s ataxia is characterized by degeneration of large sensory neurons and cardiomyopathy (Gordon, 2000), but brain atrophy and iron accumulation are also features of the disease (Synofzik et al., 2011). Recent studies suggested that the function of frataxin is related to the maintenance of iron homeostasis, acting as iron-storage protein in mitochondrial similar to ferritin, and also an intramitochondrial iron chaperone. It is also suggested to be involved in heme and iron sulfur cluster biogenesis. The frataxin mutant is unstable and severe reduction of the protein results in intramitochondrial iron accumulation and cytosolic iron deficiency in mice and humans, and is suggested to contribute to the pathogenesis of the disease (Gordon, 2000; Puccio et al., 2001; Richardson et al., 2010). Interestingly, a high iron diet limits some of the phenotypes in mouse models such as cardiac hypertrophy (Whitnall et al., 2012).

Like iron, copper also participates in neurodegenerative pathways. Copper is able to cause the aggregation of alpha synuclein (Bharathi and Rao, 2008), and copper is a co-factor of dopamine beta-hydroxylase, which is involved in dopamine synthesis (Ash et al., 1984). Copper is decreased in PD SN (Dexter et al., 1991; Ayton et al., 2012b) which might be a reason why the copper-dependent Cp protein is dysfunctional in the disease. Peripheral Cp is also depleted in Wilson’s disease, which is primarily a disorder of copper homeostasis, caused by a genetic mutation to ATP7b (Bull et al., 1993). Copper accumulates in liver and brain, along with iron (Shiono et al., 2001; Litwin et al., 2013). Why does iron also accumulate as a result of the disease? Possibly reduced Cp levels in plasma reduce iron export in liver and brain, resulting in iron accumulation. Whatever the mechanism, Wilson’s disease often presents as early-onset PD, possibly mediated by the elevation of copper and iron (Machado et al., 2006).

## TOXICITY MECHANISMS OF IRON OVERLOAD IN DISEASES

Iron can induce neurotoxicity by its ability to promote the formation of ROS, a source of oxidative stress. Elevated iron is potentially neurotoxic, indeed the direct injection of iron into the rat brain causes neurodegeneration (Ben-Shachar and Youdim, 1991), possibly via an oxidative stress pathway which initiates several apoptotic signaling pathways (Ke and Ming Qian, 2003).

Recently, a type of RAS-related cell death pathway was shown to be linked with intracellular iron levels, termed ferroptosis (Dixon et al., 2012), which could be potentially responsible for cell death seen in iron overload diseases. This type of cell death pathway shared no markers of apoptosis (e.g., caspase activation, mitochondrial cytochrome c release), but could be prevented by iron chelation or iron uptake inhibition (Yagoda et al., 2007; Yang and Stockwell, 2008). This pathway is not induced by Fenton chemistry; rather it is related with iron-dependent enzymatic activities (Dixon et al., 2012). Indeed, inappropriate intracellular iron accumulation potentially damages a number of proteins such as Ca^2^^+^-ATPase (Kaplan et al., 1997; Moreau et al., 1998), glutamate transporter (Gnana-Prakasam et al., 2009; Yu et al., 2009; Mitchell et al., 2011), Na^+^/K^+^-ATPase (Kaplan et al., 1997; Strugatsky et al., 2003), and *N*-methyl-D-aspartate (NMDA) receptor (Nakamichi et al., 2002; Munoz et al., 2011), as well as oxidizes lipid such as cholesterol (Kraml et al., 2005; Graham et al., 2010; Shinkyo and Guengerich, 2011), ceramides (Yurkova et al., 2005), and sphingomyelin (Jenkins and Kramer, 1988; Isaac et al., 2006); all of which were proposed to ultimately cause synaptic dysfunction and neuronal cell death (Mattson, 2004).

It is therefore not surprising that iron elevation observed in a number of neurodegenerative diseases, such as AD and PD, is proposed to be a key mediator in cell loss of these diseases (Ayton et al., 2012a; Lei et al., 2012). In neurodegenerative diseases, iron is also found to partner with disease-related proteins, such as β-amyloid, tau, prion, and α-synuclein, which form soluble and insoluble aggregates and activate cell death pathways (Chiti and Dobson, 2006). The presence of iron accelerates the aggregation process *in vitro *(Schubert and Chevion, 1995; Ostrerova-Golts et al., 2000; Rottkamp et al., 2001; Yamamoto et al., 2002; Khan et al., 2006), and aggravates the oxidative stress induced by the protein *in vivo *(Huls et al., 2011; Li et al., 2011; Wan et al., 2011).

Recently it has emerged that these disease-related proteins also participate in iron metabolism. The mRNA of APP has an IRE in its 5′-UTR (Rogers et al., 2002, 2008), and was found to facilitate iron export *in vitro* and *in vivo* (Duce et al., 2010). Suppression of APP expression in mice resulted in age-dependent iron accumulation (Duce et al., 2010), and overexpression of wild type APP resulted in iron reduction in SH-SY5Y neuroblastoma cells (Wan et al., 2012b). Interestingly, overexpression of a disease-related mutant form of APP, the Swedish mutant, in SH-SY5Y cells and *Caenorhabditis elegans* causes significant iron retention accompanied with elevated ROS (Wan et al., 2011). It was proposed by the authors that the observed iron change is due to the increased amount of Aβ (Wan et al., 2011), however, it can be alternatively explained by loss-of-APP function. Aβ oligomers were shown to decrease NTBI uptake, however, the disease relevance was unclear (SanMartin et al., 2012). Recently, tau protein was found to mediate APP trafficking, and reduction of tau blocked iron export, leading to intracellular iron accumulation (Lei et al., 2012). Tau knockout mice exhibited age-dependent neurodegeneration, which could be pharmacologically prevented by iron chelation therapy (Lei et al., 2012), supporting a function of tau in iron metabolism.

Other disease related proteins have been investigated less for their association with iron metabolism, but the emerging data could point to a role for these proteins in iron homeostasis. α-synuclein exhibits an IRE in its 5′-UTR mRNA (Friedlich et al., 2007), and is reported to be ferrireductase of unknown biological function (Davies et al., 2011). Recently, prion protein was also suggested to act as a functional ferrireductase, to modulate cellular iron uptake (Singh et al., 2009c, 2013). Loss of prion protein caused iron deficiency in mice, which can be reversed by expression of wild type prion protein (Singh et al., 2009b). In addition, huntingtin protein, involved in Huntington’s disease, was also reported as an iron-responsive protein (Hilditch-Maguire et al., 2000). In huntingtin-deficient zebrafish, iron starvation was identified during development, and these zebrafish had decreased hemoglobin production (Lumsden et al., 2007).

## THERAPEUTICS BASED ON IRON MODULATION

Since iron involves the pathogenesis of neurodegenerative disorders, chelation of iron therefore could be a therapeutic strategy. Currently, iron chelation is utilized in practice for transfusional iron overload and hemochromatosis (Nick, 2007). Treatment for this type of diseases requires selective iron chelators with high affinity, to facilitate bulk excretion of iron from the body (Positano et al., 2009; Meloni et al., 2010; Murphy and Oudit, 2010; Pietrangelo, 2010). Deferoxamine (Propper et al., 1976), deferiprone (Kontoghiorghes et al., 1987a, b) and deferasirox (Piga et al., 2006; Shashaty et al., 2006) have been tested for these diseases. However, neurodegenerative diseases that feature regional iron accumulation require therapeutic agents to cross blood–brain barrier, and target specific brain regions in preference to the rest of the body.

Several brain permeable iron chelators have been explored in pre-clinical models of AD and PD (Kontoghiorghes et al., 1987a; Ben-Shachar et al., 1992; Kaur et al., 2003; Youdim et al., 2004a; Liang et al., 2008; Gogoi et al., 2011) although none of these compounds have entered clinical trials so far. One pilot trial of deferiprone was reported to be beneficial for NBIA (Abbruzzese et al., 2011). The mechanisms for neuroprotection effects of iron chelators have been linked with suppression of apoptotic pathway (Youdim et al., 2005; Avramovich-Tirosh et al., 2007; Zhu et al., 2007; Amit et al., 2008; Gal et al., 2010), promoting survival pathways (Avramovich-Tirosh et al., 2010; Reznichenko et al., 2010), restoration of protein degradation (Zhu et al., 2007), and stabilization of mitochondrial function (Youdim et al., 2005).

Clioquinol is a moderate affinity iron chelator that has undergone extensive pre-clinical testing for neurodegenerative disorders, and a clinical trial (Cherny et al., 2001; Kaur et al., 2003; Ritchie et al., 2003; Lei et al., 2012). The therapeutic effects of clioquinol have often been attributed to its ionophore activity, which redistributes copper and zinc into the cell (Cherny et al., 2001; Nitzan et al., 2003; Adlard et al., 2008; Li et al., 2010; Crouch et al., 2011; Park et al., 2011). However, its ability to chelate iron is also likely involved in its neuroprotective properties. Iron binds to clioquinol (Tamura et al., 1973; Kidani et al., 1974; Ohtsuka et al., 1982), and several beneficial effects of clioquinol have reported to be iron-dependent (Felkai et al., 1999; Atamna and Frey, 2004; Choi et al., 2006; Rival et al., 2009). Treatment with clioquinol prevents the elevation of SN iron levels in MPTP-treated mice, which confers neuroprotection (Kaur et al., 2003). Similar treatment also prevented age-related nigra degeneration in tau knockout mice (Lei et al., 2012), highlighting a potential use of clioquinol as an iron-binding agent. These results suggest that clioquinol participates in iron redistribution, but more data is needed to confirm.

## CONCLUSION

The tightly regulated nature of iron in the human brain protects against diseases associated with excess or deficiency. Disease manifests when these systems deteriorate or are overwhelmed. Iron deficiency is prevalent, particularly in underdeveloped societies, and causes long-term consequences to brain health. There is therefore urgent need to address nutritional deficiency in pregnancy and in infancy to prevent these long-term consequences. Iron elevation in the brain is a feature of several major neurodegenerative disorders. While the cause of this is unknown, it is noteworthy that a variety of neurodegenerative disease-associated proteins involved in iron metabolism through various mechanisms, supporting the hypothesis that iron and disease-related proteins participate in a toxic cycle. The involvement of iron in neurodegenerative diseases needs further elucidation, but iron overload in these disorders represents an attractive pharmacological target for disease modifying therapies.

## Conflict of Interest Statement

The authors declare that the research was conducted in the absence of any commercial or financial relationships that could be construed as a potential conflict of interest.

## ABBREVIATIONS

AD: Alzheimer’s disease
ADHD: attention deficit hyperactivity disorder; apo-Tf, apo-transferrin;
ATP: adenosine-5′-triphosphate;
BBB: blood–brain barrier;
BCEC: brain capillary endothelial cells;
BID: brain iron deficiency;
cAMP: cyclic adenosine monophosphate;
Cp: Ceruloplasmin;
CREB: cyclic adenosine monophosphate response element-binding protein;
CSF: cerebrospinal fluid;
GFAP: glial fibrillary acidic protein
DMT1: divalent metal transporter 1; Fe, iron; Fe2Tf/holoTf, holo-transferrin;
GWAS: genome-wide association study;
HCP1: heme carrier protein 1;
HFE: human hemochromatosis protein;
HIF: hypoxia inducible factor;
ICP-MS: inductively coupled plasma-mass spectrometry;
IRE: iron responsive element; IRP1/2, iron regulatory proteins 1/2;
LMW: low molecular weight;
MMSE: mini-mental state examination;
MPTP: methyl-4-phenyl-1,2,3,6-tetrahydropyridine;
MRI: magnetic resonance imaging; mRNA, messenger RNA;
NBIA: neurodegeneration with brain iron accumulation;
NTBI: non-transferrin bound iron; 6-OHDA, 6-hydroxydopamine;
PD: Parkinson’s disease;
RLS: restless legs syndrome;
ROS: reactive oxygen species;
SN: substantia nigra;
STEAP: 1-4, six-transmembrane epithelial antigen of prostate 1-4;
TCS: transcranial sonography;
Tf: transferrin;
TFR1: transferrin receptor 1;
TfR2: transferrin receptor 2;
UTR: unstranslated region;
MAPK: mitogen-activated protein kinase; Erk1/2, extracellular signalregulated kinase 1/2;
GSK-3: glycogen synthase kinase 3;
Cdk-5: cyclin-dependent kinase 5;
AMPA: á-amino-3-hydroxy-5-methyl-4-isoxazolepropionic acid;
Bcl-2: B-cell lymphoma 2;
NMDA: N-methyl-D-aspartate.
